# T93. PERSONALITY TRAITS AND PSYCHOTIC SYMPTOMS IN RECENT ONSET OF PSYCHOSIS PATIENTS

**DOI:** 10.1093/schbul/sby016.369

**Published:** 2018-04-01

**Authors:** Julia Sevilla-Llewellyn-Jones, Olga Santesteban-Echarri, Pablo Cano-Domínguez, Antonia de-Luis-Matilla, Alberto Espina-Eizaguirre, Berta Moreno-Kustner, Susana Ochoa

**Affiliations:** 1 San Carlos Clínico Hospital; 2 University of Calgary; 3 Hospital Clínico Universitario Virgen de la Victoria; 4 Málaga University; 5 Hospital Sant Joan de Deu

## Abstract

**Background:**

Personality in patients with psychosis, and particularly its relation to psychotic symptoms in recent onset of psychosis (ROP) patients, is understudied. The aims of this research were, first, to study the relation between each clinically significant personality trait (CSPT) and psychotic symptoms, and second, to study the variance of each CSPT that was explained by psychotic symptoms, sex and age.

**Methods:**

Sample: Data was obtained from 94 ROP patients (78.7% males; mean age: 24.67(4.59)). Measures: The Millon Clinical Multiaxial Inventory (MCMI) and the Positive and Negative Syndrome Scale were used to assess CSPT (schizoid, avoidant, dependent, depressive, histrionic, narcissistic, antisocial, sadistic, compulsive, negativistic, masochistic, schizotypal, borderline, paranoid) and psychotic symptoms (positive, negative, disorganized, exited, and anxiety and depression). Statistical analyses: U Mann–Whitney tests and multivariate logistic regressions were carried out to test the association between clinical personality traits adjusting for symptoms, sex and age.

**Results:**

From the 94 patients, 13.9% had schizoid, 20.8% had avoidant, 7.9% had depressive, 7.9% had dependent, 5% histrionic, 15.8% narcissistic, 9.9% antisocial, 6.9 % sadistic, 14.9% compulsive, 1% negativistic, 0% masochistic, 2% schizotypal, 2% borderline, 5% paranoid CSPT. Negative psychotic symptoms were higher in patients with schizoid CSPT. The excited symptoms were lower for those with avoidant and depressive CSPT. The anxiety and depression symptoms were higher for patients with dependent CSPT. The positive psychotic symptoms were lower for patients with histrionic and higher for patients with compulsive CSPT. Logistic regression demonstrated that gender and positive and negative symptoms explained 35.9% of the variance of the schizoid CSPT. Excited symptoms explained 9.1% of the variance of the avoidant CSPT. Anxiety and depression symptoms and age explained 31.3% of the dependent CSPT. Gender explained 11.6% of the histrionic CSPT, 14.5% of the narcissistic CSPT and 11.6% of the paranoid CSPT. Finally, gender and positive dimension explained 16.1% of the compulsive CSPT.

**Discussion:**

The study highlights the importance of studying personality in patients with psychosis as it broadens understating of the patients themselves and the symptoms suffered.

